# Warm-Up Intensity and Time-Course Effects on Jump Height under Cold Conditions

**DOI:** 10.3390/ijerph19095781

**Published:** 2022-05-09

**Authors:** Itaru Chiba, Mina Samukawa, Kazuki Takizawa, Yuriko Nishikawa, Tomoya Ishida, Satoshi Kasahara, Masanori Yamanaka, Harukazu Tohyama

**Affiliations:** 1 Department of Rehabilitation, Nishioka Daiichi Hospital, Sapporo 062-0033, Japan; abc090def@gmail.com; 2 Faculty of Health Sciences, Hokkaido University, Sapporo 060-0812, Japan; t.ishida@hs.hokudai.ac.jp (T.I.); kasahara@hs.hokudai.ac.jp (S.K.); tohyama@med.hokudai.ac.jp (H.T.); 3 Institute of Physical Development Research, Sapporo 060-0061, Japan; takizawa@pd-r.org; 4 Department of Exercise Physiology, Nippon Sport Science University, Tokyo 158-8508, Japan; moose.marinos@gmail.com; 5 Faculty of Health Science, Hokkaido Chitose College of Rehabilitation, Chitose 066-0055, Japan; m-yamanaka@chitose-reha.ac.jp

**Keywords:** cold, high-intensity, warm-up, moderate-intensity, counter-movement jump, temperature

## Abstract

With this study, we aimed to investigate the effects of different warm-up intensities on counter-movement jump (CMJ) performance over time under cold conditions. Eleven male collegiate athletes volunteered. The participants performed high-intensity warm-up (HWU) at 80% VO_2max_ and moderate-intensity warm-up (MWU) at 60% VO_2max_ for 15 min on a bicycle ergometer in a laboratory room at 10 °C. CMJ height, vastus lateralis muscle temperature, heart rate, and perceived fatigue were measured before warm-up (Pre), immediately after (Post 0), 10 min after (Post 10), and 20 min after (Post 20). Significant main effects and interactions were found for CMJ height (time, *p* < 0.001 and η_p_^2^ = 0.859; interaction, *p* = 0.007 and η_p_^2^ = 0.327). HWU significantly increased CMJ height at Post 0 to Post 20 compared to that at Pre (*p* < 0.01), whereas MWU increased CMJ height at Post 0 only compared to that at Pre (*p* < 0.001). The results indicate that HWU achieved an increase in CMJ height for 20 min. MWU changed CMJ height instantly, but the change did not last compared to HWU in a cold environment.

## 1. Introduction

Warm-up before exercise or competition is commonly used in preparing for athletic demands and for injury prevention [1]. A recent meta-analysis reported that warm-up effectively enhances performance [2]. Structural warm-up routines were reported to reduce lower limb injuries [3,4]. Therefore, warm-up is essential in performance enhancement and injury prevention.

Warm-up is usually performed by targeting four physiological phases (raise, activate, mobilise, and potentiate) and sport-specific drills [5,6,7]. The “raise phase” increases muscle and body core temperatures. It has been shown that increased muscle temperature positively affects physical performance [5,7,8,9]. The power output of pedalling with fast and slow velocity improved by 4.9% and 4.2%, respectively, for every 1 °C increase in muscle temperature [9]. Vertical jump height increased by 4.0%, whereas knee-extensor isometric torque increased by 2.1% for every 1 °C increase in muscle temperature [8]. With a 1 °C increase in muscle temperature, short-duration exercise performance improved from 2 to 5% [10]. Therefore, increasing muscle temperature with a warm-up is key to potentiating dynamic movement before competitions or training. In a previous study by Bishop [5], a 60% VO_2max_ intensity warm-up was found to optimal to improve the performance of short-term tasks with maximal effort. Tsurubami et al. [11] demonstrated that a 60% VO_2max_ warm-up improved jump height immediately after warm-up but did not increase jump height 10 and 20 min after warm-up. Nevertheless, jump height after an 80% VO_2max_ intensity warm-up increased immediately and 10 and 20 min after the warm-up compared to that before warm-up. Furthermore, jump height after 80% VO_2max_ was higher than that after 60% VO_2max_ 20 min after the warm-up. Based on the abovementioned information, it is important to understand the effects of warm-up intensities. It is also important to consider the duration of warm-up. According to a review by Bishop’s [5], a time interval of 5–15 min after warm-up is recommended for energy system resynthesis. Although a consensus has not been reached, warm-up is beneficial and adversely influences subsequent performance, depending on the intensity, duration, and transition time. Low-intensity warm-up may lead to an insufficient increase in muscle temperature and a decreased effect on enhancing dynamic movement [12,13].

Neuromuscular function is impaired with decreased body temperature in cold environments [10]. Drop vertical jump and throwing performance are impaired, and the relationship of agonist and antagonist muscle activation with a decrease in body temperature is related to poor performance [14,15]. Warm-up is important for high-performance enhancement with increasing muscle temperature in cold environments. 

Increased muscle temperature is important in cold environments; however, the exact effects are not well described. After cold exposure at 10 °C for 60 min, a 15 min treadmill walk did not increase muscle temperature or jump performance [16]. It is necessary to investigate the effects of warm-up intensity on muscle temperature and athletic performance in cold environments.

Accordingly, with this study, we aimed to examine the time-course effects of warm-ups performed at two different intensities on jump performance and muscle temperature during cold exposure. We hypothesised that both high-intensity and moderate-intensity warm-ups could improve jump performance. Furthermore, jump performance could be improved more with HWU than with MWU, as the former is expected to induce a greater increase in muscle temperature than the latter.

## 2. Materials and Methods

### 2.1. Study Design

**Figure 1:**
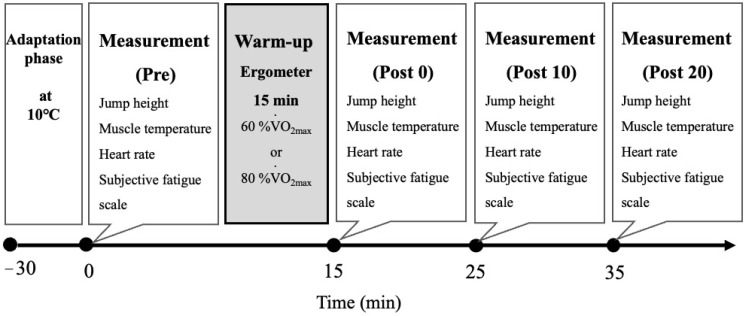
Study design.

This was a randomised crossover study. An outline of the study is shown in Figure 1. After participants came into the laboratory room, they sat on a chair and rested for 30 min in the same long-sleeved T-shirt and shorts throughout the experiment to adapt to the laboratory temperature. On the first day, an incremental exercise test, physical assessment, and practice of counter-movement jump (CMJ) were conducted as a familiarisation trial. On the second and third days, all participants performed either a moderate-intensity warm-up (MWU) or a high-intensity warm-up (HWU) in a random order. Muscle temperature, CMJ, heart rate, and subjective fatigue level were measured before warm-up (Pre) and immediately (Post 0), 10 min (Post 10), and 20 min after warm-up (Post 20). The laboratory temperature was set at 10 °C.

### 2.2. Participants

Eleven male collegiate athletes practicing sports regularly (age, 20.8 ± 1.0 years; height, 173.9 ± 4.3 cm; weight, 67.9 ± 4.7 kg; body mass index, 22.5 ± 1.1 kg/m^2^; and maximum oxygen uptake, 54.7 ± 5.0 mL/min) participated. Participants with any orthopaedic or neurological diseases were excluded from the study. Participants with a body mass index <18.5 or >25.0 kg/m^2^ were also excluded because body composition affects temperature changes [17]. Participants were instructed to avoid intensive exercises or drinking alcohol for 24 h before the test period and to not eat meals 2 h before the experimental session. All participants performed each test at the same time of day. The second day of testing was a week after the incremental exercise test to avoid fatigue. The second and third days of the study were at least two days apart. Participants wore the same long-sleeved T-shirt, shorts, and shoes during all experiments. All participants were verbally informed of the study purposes, protocols, and risks with a written document, and they signed a consent form. The ethical committee of the Faculty of Health Sciences of Hokkaido University approved this study (approval number: 17–103).

### 2.3. Determination of Warm-Up Intensity

During incremental exercise, all participants performed a bicycle ergometer test (POWER-MAX V; KONAMI, Tokyo, Japan). The test was initiated at 60 W, followed by a 30-W increase every 3 min and a 1 min rest until the participant was unable to maintain a cadence of 60 repetitions per minute (RPM). The incremental exercise test was completed when the oxygen uptake level reached a plateau, more than 1.1 of the respiratory quotients, or when the participant was unable to pedal anymore. Oxygen uptake was measured using a respiratory gas analyser (AE–280, MINATO MEDICAL SCIENCE Co., Ltd., Tokyo, Japan) every 10 s, and 30 s averaged data were obtained to determine the warm-up intensity. The highest value during incremental exercise was defined as the VO_2max_, and the exercise intensities at 60 and 80% VO_2max_ were determined based on the relationship between intensity and oxygen uptake. Warm-up was performed by riding on a bicycle ergometer for 15 min at 60 RPM. Pedalling cadence at 60 RPM was recommended for low-to-average fitness populations [18,19]. In this study, MWU was at 178.2 ± 15.6 W, and HWU was at 242.4 ± 21.6 W.

### 2.4. Measurements

The vastus lateralis (VL) muscle temperature of the dominant leg was measured using a core body thermometer (Core temp CTM-205, Terumo, Tokyo, Japan). Heart rate (HR) was assessed using a portable heart rate monitor (RCX5, Polar, Kimpel, Finland). A visual analogue scale (VAS) was used to assess subjective fatigue levels. VAS consisted of a 100 mm horizontal line labelled “no fatigue” on the left end and “maximal fatigue and cannot walk even a step” on the right end [20]. For performance measurements, CMJ heights were estimated using a force plate (Ex-Jumper, DKH, Tokyo, Japan). Jump heights were calculated from a force–time curve.

$$\mathrm{Jump}\;\mathrm{height}=\frac{1}{8}\times g\times {\left(\mathrm{flight}\;\mathrm{time}\right)}^{2}$$

Participants performed CMJs with both hands on their hips. They were verbally encouraged to jump high with maximum effort. CMJ was performed three times, and the highest jump in each interval was used in the analysis. 

### 2.5. Statistical Analysis

Statistical analyses were performed using SPSS version 22 software (IBM, Chicago, IL, USA). The Shapiro–Wilk test was conducted for normality. A two-way analysis of variance (intensity [HWU and MWU] × time [Pre, Post 0, Post 10, and Post 20]) was used to compare variations in HR, VAS, muscle temperature, and CMJ heights. Bonferroni correction was used as a post-hoc test. A *p*-value < 0.05 was considered statistically significant. For the effect size, partial eta-squared values (η^2^_p_) for repeated measures were calculated.

## 3. Results

### 3.1. CMJ Height

Significant interaction effects (intensity × time) for CMJ height were found (time, *p* < 0.001 and η^2^_p_ = 0.859; and interaction, *p* = 0.007 and η^2^_p_ = 0.327), although intensity did not have a significant effect (*p* = 0.33 and η^2^_p_ = 0.094). A post-hoc test revealed that CMJ heights at Post 0, Post 10, and Post 20 were significantly higher than those at Pre with HWU (*p* < 0.001–0.01). However, as shown in Table 1, CMJ height was higher with MWU only at Post 0 than at Pre (*p* < 0.001). A post hoc test revealed that a significant difference was found at Post 20 between HWU and MWU (*p* = 0.021).

### 3.2. Muscle Temperature

There were significant effects of intensity, time, and interaction (intensity × time) on VL muscle temperature (time, *p* < 0.001 and η^2^_p_ = 0.975; intensity, *p* = 0.004 and η^2^_p_ = 0.573; and interaction, *p* < 0.001 and η^2^_p_ = 0.625). There was a significant increase in muscle temperature from Pre to Post 0, Post 10, and Post 20 with both HWU and MWU (*p* < 0.001) (Table 1). A post-hoc test revealed that muscle temperature with HWU was significantly higher at Post 0, Post 10, and Post 20 than that with MWU (*p* < 0.001–0.007) (Table 1).

### 3.3. Heart Rate

Significant effects of intensity, time, and interaction (intensity × time) on HR were observed (intensity, *p* < 0.001 and η^2^_p_ = 0.844; time, *p* < 0.001 and η^2^_p_ = 0.988; and interaction, *p* < 0.001 and η^2^_p_ = 0.730). A post-hoc test revealed a significant increase in HR at Post 0, Post 10, and Post 20 compared to that at Pre with HWU (*p* < 0.001) (Table 1). The HRs in MHU were significantly higher at Post 0 and Post 10 than those at Pre (*p* < 0.001 and *p* = 0.002, respectively). The HRs in HWU were significantly higher than those in MWU at Post 0, Post 10, and Post 20 (*p* < 0.001–0.017) (Table 1).

### 3.4. Subjective Fatigue Scale

Significant effects of time and interaction (intensity × time) were found for the subjective fatigue level (time, *p* < 0.001 and η^2^_p_ = 0.846; interaction, *p* = 0.004 and η^2^_p_ = 0.355), yet intensity did not have any main effects (intensity, *p* = 0.057 and η^2^_p_ = 0.316). In the HWU group, subjective fatigue scale scores at Post 0 and Post 10 were higher than those at Pre (*p* < 0.001 and *p* = 0.042, respectively). With MWU, the scores of subjective fatigue at Post 0 and Post 10 were higher than those at Pre (*p* = 0.001 and *p* = 0.014, respectively) (Table 1).

## 4. Discussion

This study revealed the time-course effects of two warm-ups with different intensities on CMJ performance under cold temperatures. HWU was more effective for time-course effects on jump performance than MWU in a cold environment.

As for the results of the comparison of Post 0 with Pre, muscle temperature, HR, and subjective fatigue level with HWU were significantly higher than those with MWU. After HWU and MWU, jump height was significantly higher at Post 0 than at Pre, and there was no significant difference between HWU and MWU. The increases in muscle temperature with short-term exercise had a positive effect on jump height at Post 0 [8]. Muscle temperature was significantly higher after HWU than after MWU. Moreover, there were significant differences in HR and subjective fatigue levels between the HWU and MWU groups. Previous studies have reported that intermuscular pH decreases and muscle contraction is inhibited after intensive exercise, and athletic performance is negatively affected by fatigue [21,22,23]. Subjective fatigue was significantly higher with HWU than with MWU at Post 0 only, and the results of this study showed that there were no increments in CMJ height between HWU and MWU. The influence of subjective fatigue on jump performance warrants further investigation. 

Muscle temperature and HR at both Post 10 and Post 20 were significantly higher with HWU than with MWU. Intensity did not have any significant effects on jump height. If an athletic event is held immediately after warm-up under cold conditions, MWU is considered more beneficial than HWU because it produces the same jump performance with less effort than the latter. Moreover, our results indicate that HWU is more effective in maintaining the increase jump height for a longer duration than MWU. After HWU, CMJ height was significantly higher at Post 10 and Post 20 compared with that at Pre, yet there were no significant differences between Post 10 or Post 20 and Pre with MWU. Jump height maintained an increase of approximately 6.4 % (2.3 cm) after HWU and an increase of 2.0 % (0.7 cm) after MWU at Post 20. 

Under thermoneutral conditions, jump height at Post 20 was significantly higher with HWU than with MWU [11]. Additionally, previous studies have shown that muscle temperature is significantly higher after HWUs than after MWUs. Exercise intensity was related to larger increments of body temperature; however, high muscle temperature increments after high-intensity exercise and diminishing fatigue lead to high CMJ heights with HWU [11]. Other studies showed that HWU causes poor performance, owing to fatigue [22,23], and that negative influences disappear over time [24]. Therefore, HWU can improve jump performance if there are 10–20 min between WUP and competition, even in a cold environment.

In this study, we defined a cold environment as a temperature of 10 °C based on previous studies [14,15,16,25]. In winter sports, warm-up is often conducted in a cold environment below 10 °C. Previous studies conducted at 19.5 °C and 10 °C indicated that wearing extra clothes maintains muscle temperature and avoids a drop in muscle temperature when exposed to cold [26,27]. After 25 min warm-up procedures including jogging, sprinting, dynamic stretching, plyometrics, and passive heat maintenance were conducted, the decline in core body temperature and CMJ power output were effectively attenuated [26]. Covering legs during cold exposure has been shown to prevent a decline in jump performance [27]. This may be sufficient to enhance performance with MWU and additional clothing. Furthermore, previous research has shown that a decrease in body temperature may decrease the lactate threshold and maximal oxygen uptake with cold exposure [25,28,29]. This study result is also similar to that reported in a previous study, which indicated that HWU diminished fatigue within 10–20 min after warm-up [11]. Metabolic or cardiovascular changes in a more severe cold environment may contribute to anaerobic metabolism during warm-up, and fatigue is more likely to occur in cold environments than in a thermoneutral environment. Therefore, the relationship between warm-ups of different intensities under various cold conditions must be investigated.

Warm-ups in sports consist of a general warm-up phase (submaximal aerobic exercise to focus on physiological change) and differ depending on the protocol. A previous study suggested structuring progressive warm-up intensities (50–90% of maximal heart rate) and ending the warm-up with a high-intensity sprint (>90% of maximal heart rate) [7]. A higher warm-up volume may lead to cumulative fatigue, subsequently decreasing performance. Especially in team sports, decreasing warm-up intensity, duration, and total volume can cause less fatigue and positively affect subsequent athletic performance. Furthermore, stretching and sport-specific exercise drills are commonly included in warm-up routines, followed by a general warm-up subphase [30,31]. Warm-ups increase body temperature and improve stretching effects on range of motion (ROM) [32]. Specific exercises vary depending on the sport being played [22]. Thus, it is worth noting that determining the effects of warm-up on improving ROM will help establish an optimal protocol for not only injury prevention but also performance enhancement during cold exposure. 

This study has a few limitations. First, we did not set a thermoneutral condition; therefore, we were unable to compare cold and thermoneutral environments. Secondly, there were no other parameters regarding thermoregulation (i.e., body core temperature). Finally, the sports background of participants was not unified; therefore, the effects of warm-up may have been different, and the different ages, levels, and sports of participants may have affected the results. 

The present research suggests several practical applications, as indicated below:

- If an athletic event is held immediately after warm-up under cold conditions (10 °C), athletes should use moderate-intensity warm-up (60% VO_2max_) because it produces the same jump performance with less effort than high-intensity warm-up.
- High-intensity warm-up (80% VO_2max_) is recommended when athletes have a brief time interval between warm-up and the start of an athletic event under cold conditions.

## 5. Conclusions

We investigated the effects of warm-ups at two different intensities (HWU and MWU) on jump height, muscle temperature, heart rate, and subjective fatigue level under cold conditions. Our results demonstrate that both HWU and MWU led to an increase in jump height immediately after warm-up, yet there were no differences in jump height between HWU and MWU. The increase in jump height was maintained for 20 min after HWU only, and the jump height was significantly higher after HWU than after MWU at Post 20. Muscle temperature was significantly higher at Post 0, Post 10, and Post 20 than at Pre with both warm-ups. Moreover, muscle temperature and heart rate were higher at Post 0, Post 10, and Post 20 with HWU than with MWU. The present study findings reveal HWU to improve jump performance and maintain it for 20 min. HMU is recommended when there is a brief interval before an athletic event.

## Figures and Tables

**Table 1:** Time-course changes in CMJ height, muscle temperature, heart rate, and subjective fatigue scale.

		Pre	Post 0	Post 10	Post 20	Interaction
CMJ height (cm)	HWU	35.8 ± 3.9	41.1 ± 4.4 *	39.3 ± 3.9 *	38.1 ± 3.7 *^#^	*p* = 0.007
	MWU	36.0 ± 3.5	41.7 ± 3.5 *	37.7 ± 3.9	36.7 ± 3.4	
Muscle temperature (°C)	HWU	33.6 ± 0.5	37.5 ± 0.4 *^#^	37.2 ± 0.2 *^#^	36.6 ± 0.2 *^#^	*p* = 0.004
	MWU	33.6 ± 0.5	36.4 ± 0.6 *	36.4 ± 0.7 *	36.0 ± 0.6 *	
Heart rate (beats/min)	HWU	65.7 ± 4.4	171.6 ± 6.2 *^#^	99.7 ± 6.2 *^#^	83.6 ± 8.2 *^#^	*p* < 0.001
	MWU	64.4 ± 5.6	131.8 ± 5.1 *	82.4 ± 7.9 *	70.0 ± 6.9	
Subjective fatigue scale (mm)	HWU	17.0 ± 10.8	82.0 ± 12.8 *^#^	47.3 ± 23.5 *	44.7 ± 21.9	*p* = 0.004
	MWU	20.6 ± 18.0	56.8 ± 18.9 *	33.3 ± 10.5 *	29.0 ± 12.1	

Data are presented as mean ± SD. HWU, high-intensity warm-up; MWU, moderate-intensity warm-up; Pre, before warm-up; Post 0, immediately after warm-up; Post 10, 10 min after warm-up; Post 20, 20 min after warm-up; * significantly higher compared to Pre; # significantly higher with HWU than MWU.

## Data Availability

The datasets in this study are available upon reasonable request to the corresponding author’s e-mail.
